# Impact of atrial arrhythmias after esophagectomy on recovery: A meta-analysis

**DOI:** 10.1097/MD.0000000000010948

**Published:** 2018-06-18

**Authors:** Lai-Te Chen, Chen-Yang Jiang

**Affiliations:** a Zhejiang University; b Sir Run Run Shaw Hospital, Zhejiang University, School of Medicine, Hangzhou, China.

**Keywords:** anastomotic leakage, esophagectomy, length of stay, meta-analysis, mortality, postoperative atrial arrhythmias, pulmonary pneumonia

## Abstract

**Background:**

Postoperative atrial arrhythmias (PAAs) are common complications after esophagectomy, however research findings are contradicted on the prognosis. Therefore this meta-analysis was conducted to determine whether PAAs after esophagectomy had an impact on prognosis.

**Methods:**

**S**tudies comparing prognosis between patients with and without PAAs after esophagectomy were searched in EMBASE, MEDLINE, and the Cochrane Register. Primary prognosis was perioperative mortality, and secondary prognoses were postoperative complications, length of stay (LOS).

**Results:**

Ten studies including 2681 patients were included in this analysis, in which 508 patients (18.9%) experienced PAAs. Patients with PAAs resulted in significantly higher perioperative (odds ratio, OR 4.05[95% confidence interval, CI: 2.45–6.70], *P* = .40) mortality, longer hospital LOS (mean differences, MD: 1.49 [95% CI: 0.32–2.66]days, *P* = .01), more incidence of pulmonary pneumonia (OR 2.48 [95% CI: 1.71–3.59], *P* < .00001), and anastomotic leakage (OR 2.37 [95% CI: 1.39–4.03], *P* < .00001).

**Conclusions:**

Atrial arrhythmias (AAs) after esophagectomy are associated with higher perioperative mortality, longer hospital LOS, and more incidences of complications. Therapeutic strategies against PAAs are pending for further researches.

## 1 Introduction

Atrial arrhythmias (AAs), mainly atrial fibrillation, after esophagectomy are common complications.^[1]^ But the clinical significance of postoperative atrial arrhythmias (PAAs) after esophagectomy are under dispute.^[2–11]^ Many authors have conducted research about the impact of PAAs on complications,^[2–9]^ mortality,^[2,3,5–11]^ and length of stay (LOS)^[3,5–11]^ after esophagectomy, but no unanimous conclusion has been reached. Many studies demonstrate that there is an increased incidence of complication,^[3–5,9]^ length of hospital stay,^[3,5,7,10]^ as well as mortality rate^[2,8–10]^ in patients with AAs after esophagectomy, while others^[6,11]^ suggest otherwise. We conducted this meta-analysis to decide whether AAs after esophagectomy hamper recovery.

## 2 Materials/Methods and search strategy

This analysis was conducted utilizing the PRISMA flow diagram.^[12]^ The studies in English, containing data with the comparison of the prognosis between patients with and without AAs after esophagectomy were extracted from MEDLINE, EMBASE, and the Cochrane Register of controlled trials. PAAs were defined as atrial fibrillation (AF), atrial flutter (AFL), or atrial tachycardia within 30 days after esophagectomy. Considering automatic recognition of synonyms in information retrieval from the database, we used following keywords in the search: “atrial fibrillation,” “atrial flutter,” “atrial arrhythmias,” “atrial tachycardia,” and “esophagectomy.” Furthermore, references of all articles were manually searched to retrieve extra relevant data. Articles of conference and case reports were excluded. The date of the last search was 01/11/2017. Studies search was processed independently by 2 authors. A third reviewer would make the judgment if different opinions appeared between 2 reviewers. We excluded the David Amar 2002 study from the analysis of mortality, LOS, and complications due to the reason that this literature enlisted several procedures other than esophagectomy. The request for the original data was sent to the corresponding author of the literature. All analyses were based on previous published studies, thus no ethical approval and patient consent are required.

### 2.1 Primary and secondary prognosis

The primary prognosis was set as perioperative mortality, and secondary prognoses were 60-day mortality, postoperative complications (pulmonary pneumonia, renal failure, and anastomotic leakage), hospital, and intensive care unit (ICU) LOS.

### 2.2 Collection of data

Data including number of patients, stage of the disease, preoperative pulmonary function test, type of the surgery, proportion of patients with AAs after esophagectomy, onset range of PAAs, perioperative mortality, 60-day mortality, hospital LOS, ICU LOS, postoperative complication, and therapeutic strategies.

### 2.3 Assessment of quality of evidence

No evaluated scale had been used during the data gathering due to the inherent challenge nature of observational studies.^[13]^

### 2.4 Statistical analysis

Analyses were carried out with the RevMan 5.3. Continuous and dichotomous data were analyzed separately in mean differences (MD) with 95% confidence interval (CI) and odds ratio (OR) with 95% CI. For data was listed in median and interquartile range, if failure to obtain the original data from author, mean and standard deviation were calculated basing on formulas provided by Hozo et al.^[14]^ Considering heterogeneity between multi-studies, data were processed using the random effects model and were plotted with forest plot. A *P* < .05 was considered significant. Statistical heterogeneity was evaluated by the *I*^2^ test, with an *I*^2^ > 50% considering heterogeneity. Since the number of enrolled studies in this meta-analysis were relatively small, approaches for detecting publication bias would have exhibited limited efficacy, therefore, publication bias was not assessed.

## 3 Result

The disposition of searched publications was based on the PRISMA flow diagram. The time span of the studies included in this research was more than 30 years (from 1982–2016). Ten studies^[2–11]^ (Table 1)were included in our final analysis, yielding a total of 2681 patients, among which 508 patients had PAAs, giving an total incidence of 18.9%. Three studies^[4,8,11]^ examined the timeframe of PAAs, according to which, most of PAAs happened within 3 days after esophagectomy (130/145 = 89.70%). In 8 studies,^[2–6,8,9,11]^ PAAs were defined as new-onset atrial fibrillation after esophagectomy with a combined incidence of 31.8% (487/1532). Patients with prior history of AF accounted for 7.4%(9/121) and 6.5%(7/107) in R. W. Day and David Amar study respectively. Two studies^[5,10]^ with the same corresponding author from the same institution had overlapping time interval (1990–1999 vs 1995–1996). Given the potential for overlapping patients in these studies, we performed sensitivity analyses on perioperative mortality and hospital LOS after eliminating the former study.^[5]^ One study^[3]^ divided the postoperative AF into early-onset (that occur within 3 days post-operatively) and late-onset (that occur after 3 days), found no difference in complication, hospital LOS. Risk factors for PAAs included increased age,^[2,8,10]^ cardiac disease,^[2]^ amount of blood loss,^[2]^ limited intrathoracic dissection,^[2]^ thyroid disorder,^[3]^ transthoracic approach,^[3]^ presence of non-AF severe post-operative complications,^[3]^ the preoperative calcium channel blocker (CCB) medication.^[8]^

**Table 1:**
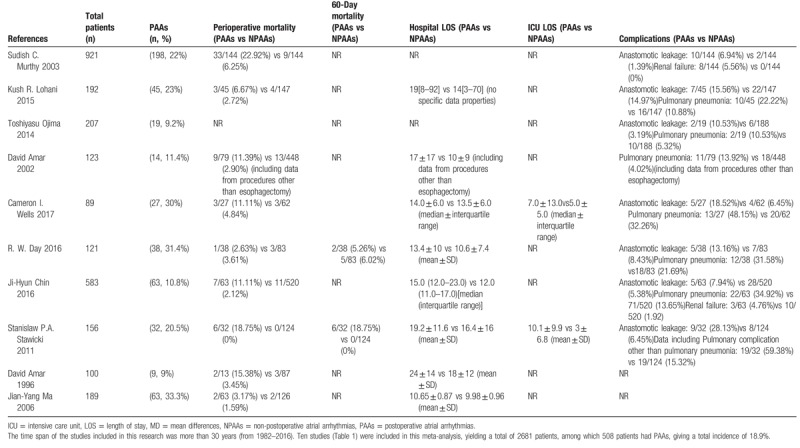
Basic characteristics of included studies.^[2–11]^.

### 3.1 Perioperative characteristic

Three studies^[2,4,8]^ characterized the TNM stage (346 patients had TNM stage III and stage IV, 587 patients had TNM stage I and stage II). Two studies^[6,8]^ characterized the preoperative ASA grade, 42 (6.9%), 537 (88.3%), and 29 (4.8%) patients were in ASA score I, II, and III, respectively. Three studies^[2,4,6]^ divided patients into subgroups base on the anatomic location of the tumor: the tumor was located in upper third of the thorax in 36 patients; in middle third of the thorax in 333 patients; in lower third of the thorax in 103 patients.^[2,4]^ And in Cameron I. Wells study, the tumor was located in middle esophagus in 15 patients; in distal esophagus in 74 patients.^[6]^ Three studies^[3,8,9]^ compared the frequency of PAAs between minimal invasive and open chest procedure and found minimally invasive transthoracic esophagectomy was not associated with lower risk of PAAs compared to open transthoracic esophagectomy. Pathology of the esophageal tumor were reviewed in 6 studies,^[2–4,6–8]^ among which squamous cell carcinoma accounted for 71.4%(1056/1422) of patients while adenocarcinoma accounted for 24.7%(366/1480). Strategies of treatment varied significantly on 8 studies^[3–5,8–11]^: temporary pacing, cardioversion ^[3]^; landiolol hydrochloride as the first-line therapy ^[4]^; digoxin, landiolol, verapamil, pilsicainide ^[5]^; electrical cardioversion or amiodarone for rhythm conversion ^[8]^; amiodarone, β-blocker, diltiazem ^[9]^; diltiazem ^[10]^; and cedilanid, isoptin, propafenone, and amiodarone.^[11]^

### 3.2 Mortality

**Figure 1:**
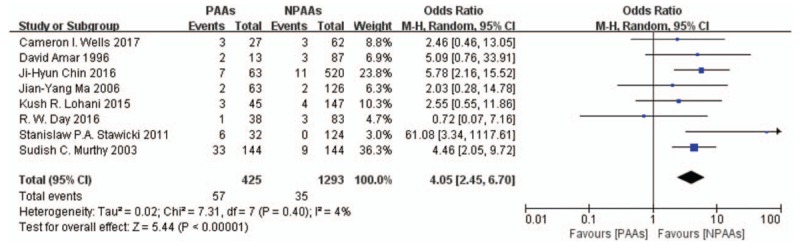
Eight studies^[2,3,6–11]^ containing 1718 patients checked perioperative (in-hospital or 30-day) mortality (92/1718 patients, incidence = 5.36%). Patients with PAAs had higher perioperative mortality (OR 4.05[95% CI: 2.45–6.70], *P* = .40, *I*^*2*^ = 4%). CI = confidence interval, OR = odds ratio, PAAs = postoperative atrial arrhythmias.

**Figure 2:**
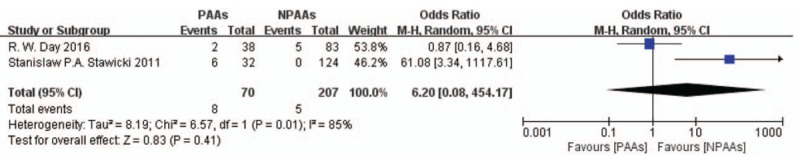
Two studies^[6,8]^ including 277 patients examined 60-day mortality (13/277 patients, incidence = 4.69%). The impact of patients with PAAs on 60-day mortality didn’t reach definitive conclusion (OR 6.20[95% CI: 0.08–454.17], *P* = .01, *I*^*2*^ = 85%). CI = confidence interval, OR = odds ratio, PAAs = postoperative atrial arrhythmias.

Eight studies^[2,3,6–11]^ containing 1718 patients checked perioperative (in-hospital or 30-day) mortality (92/1718 patients, incidence = 5.36%), while 2 studies^[6,8]^ including 277 patients examined 60-day mortality (13/277 patients, incidence = 4.69%). Patients with PAAs had higher perioperative mortality (OR 4.05[95% CI: 2.45–6.70], *P *= .40, *I*^*2*^ = 4%) (Fig. 1) but no definitive conclusion on 60-day mortality (OR 6.20[95% CI: 0.08–454.17], *P* = .01, *I*^*2*^ = 85%) (Fig. 2). Seven studies^[2,3,6–11]^ including 1597 patients compared the perioperative mortality (88/1597 patients, incidence = 5.51%) between patients with or without new-onset PAAs. Patients with new-onset PAAs had significantly higher perioperative mortality (OR 4.42[95% CI: 2.70–7.24], *P* = .54, *I*^*2*^ = 0%). After elimination of the David et al study,^[10]^ in which the PAAs defined as atrial flutter (AFL) and AF (3 patients vs 10 patients), 6 studies^[2,3,6,8,9,11]^ including 1497 patients compare the perioperative mortality (83/1497 patients, incidence = 5.54%) between patients with or without new-onset postoperative AF, Patients with new-onset postoperative AF had significantly higher perioperative mortality (OR 4.37[95% CI: 2.62–7.31], *P* = 0.41, *I*^*2*^ = 1%).

### 3.3 Length of stay

**Figure 3:**
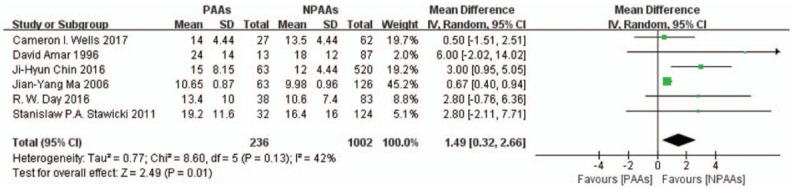
Six studies^[6–11]^ including 1238 patients examined hospital LOS. Patients with PAAs had longer hospital LOS (MD: 1.49 [95% CI: 0.32–2.66] days, *P* = .01, *I*^*2*^ = 42%). CI = confidence interval, LOS = length of stay, MD = mean differences, PAAs = postoperative atrial arrhythmias.

**Figure 4:**
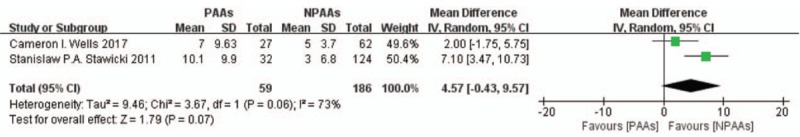
Two studies^[6,9]^ including 245 patients examined ICU LOS. No definitive conclusion was reached (MD 4.57 [95% CI:–0.43 to 9.57] days, *P* = .07, *I*^*2*^ = 73%). CI = confidence interval, ICU = intensive care unit, LOS = length of stay, MD = mean differences.

Six studies^[6–11]^ including 1238 patients examined hospital LOS. Patients with PAAs had longer hospital LOS (MD: 1.49 [95% CI: 0.32–2.66] days, *P* = .01, *I*^*2*^ = 42%) (Fig. 3). We eliminated Kush R. Lohani study^[3]^ from the analysis of hospital LOS, since no specific data property was illustrated in this article. Two studies^[6,9]^ including 245 patients examined ICU LOS. No definitive conclusion was reached (MD 4.57 [95% CI:–0.43 to 9.57]days, *P* = .07, *I*^*2*^ = 73%) (Fig. 4). After eliminating the Day et al study^[7]^ to focus strictly on new onset PAAs, 5 studies^[6,8–11]^ including 1117 patients examined hospital LOS and found that patients with new onset PAAs had longer hospital LOS (MD: 1.37 [95% CI: 0.13–2.62] days, *P* = .03, *I*^*2*^ = 45%). Four studies^[6,8,9,11]^ targeting solely on AF, revealed that patients with postoperative AF had longer hospital LOS (MD: 1.23 [95% CI: 0.05–2.41]days, *P* = .04, *I*^*2*^ = 47%).

### 3.4 Postoperative complications

**Figure 5:**
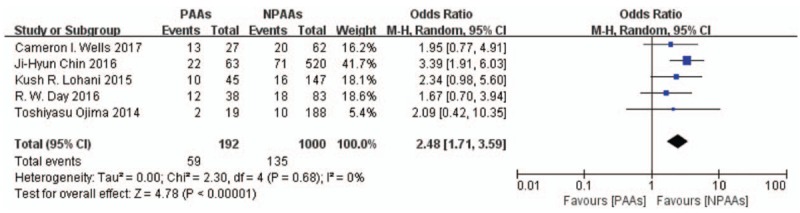
The proportion of patients with pulmonary pneumonia being the most commonly recorded complication (194/1192 patients, incidence = 16.28%). Patients with PAAs had higher risk of pulmonary pneumonia (OR 2.48 [95% CI: 1.71–3.59], *P* < .00001, *I*^*2*^ = 0%). CI = confidence interval, OR = odds ratio, PAAs = postoperative atrial arrhythmias.

**Figure 6:**
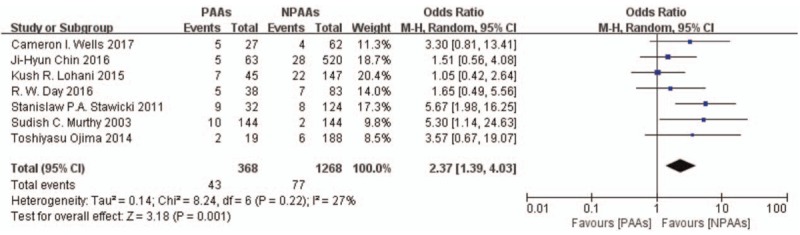
Five studies^[3,4,6–8]^ including 1192 patients examined the risk of anastomotic leakage. Patients with PAAs had higher risk of anastomotic leakage (OR 2.37 [95% CI: 1.39–4.03], *P* = .001, *I*^*2*^ = 27%). CI = confidence interval, OR = odds ratio, PAAs = postoperative atrial arrhythmias.

**Figure 7:**
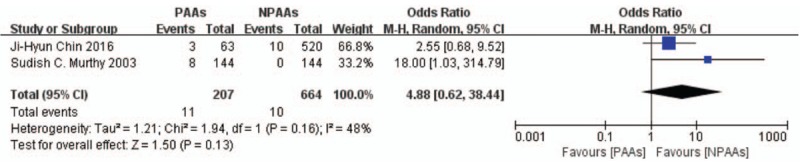
Two studies^[2,8]^ including 871 patients examined the incidence of renal failure. No definitive conclusion of the correlation between PAAs and renal failure was reached (OR 4.88[95% CI: 0.62–38.44], *P* = .13, *I*^*2*^ = 48%). CI = confidence interval, OR = odds ratio, PAAs = postoperative atrial arrhythmias.

Seven studies^[2–4,6–9]^ including 1636 patients characterized the postoperative complications, with the proportion of patients with pulmonary pneumonia being the most commonly recorded complication (194/1192 patients, incidence = 16.28%). Patients with PAAs had higher risk of pulmonary pneumonia (OR 2.48 [95% CI: 1.71–3.59], *P* < .00001, *I*^*2*^ = 0%) (Fig. 5). Five studies^[3,4,6–8]^ including 1192 patients examined the risk of anastomotic leakage. Patients with PAAs had higher risk of anastomotic leakage (OR 2.37 [95% CI: 1.39–4.03], *P* = .001, *I*^*2*^ = 27%) (Fig. 6). Two studies^[2,8]^ including 871 patients examined the incidence of renal failure. No definitive conclusion of the correlation between PAAs and renal failure was reached (OR 4.88[95% CI: 0.62–38.44], *P* = .13, *I*^*2*^ = 48%) (Fig. 7). Eliminating the Day et al study^[7]^ to focus on new onset AF after esophagectomy, revealed that patients with new onset postoperative AF had increased incidence of pulmonary pneumonia (OR 2.71 [95% CI: 1.80–4.09], *P* < .00001, *I*^*2*^ = 0%) and anastomotic leakage (OR 2.56 [95% CI: 1.38–4.76], *P* = .003, *I*^*2*^ = 37%).

## 4 Discussion

To the best of our knowledge, this is the first meta-analysis targeting the impact of PAAs on mortality, hospital LOS and complications after esophagectomy. Ten studies^[2–11]^ with 2681 patients were included in the meta-analysis, 508 among which (18.9%) experienced PAAs, mainly AF. Patients with PAAs In this meta-analysis, were tied to higher perioperative mortality; longer hospital and ICU LOS; more complications such as pulmonary pneumonia and anastomotic leakage.

Other than advanced age, the risk factors of PAAs after esophagectomy varied among studies, including history of cardiac disease,^[2,11]^ blood loss, limited intrathoracic dissection, complications with pulmonary disease and surgical sepsis^[1]^; other cardiac arrhythmia^[5]^; thyroid disfunction, procedure through thoracic^[2]^; postoperative hypoxia, history of COPD^[11]^; surgical construction with colon conduit^[4]^; medication as ccb,^[7]^ theophylline.^[9]^ Risk factors such as history of cardiac disease^[2,11]^ and other cardiac arrhythmia^[6]^ suggested that vulnerable atrial substrate may contribute to the AAs after esophagectomy.^[15]^ Studies^[3,10]^ enlisted preoperative pulmonary function as one of pre-operative variables, in which only diffusing capacity of carbon monoxide (DLCO) was associated with higher incidence of AAs, indicating hypoxia caused by low oxygen diffusion could be the trigger for atrial arrhythmias.^[16]^ Whether preoperative intervention of pulmonary strategies and medications like statin, targeting cardiac substrate could decrease the incidence of PAAs after esophagectomy, pending for approval.

The pathologic stage and histology of the tumor were illustrated in studies^[2–4,6–8]^ and showed no statistical difference between patients with and without PAAs. Hence, the disease itself might not play a vital part in triggering AAs after esophagectomy. According to studies,^[6,7,8,10]^ the effect that chemotherapy had on PAAs didn’t reach statistical significance, made the chemotherapy not likely to be the potential iatrogenic cause of AAs after esophagectomy, which was contrary to previous article.^[17]^ Published researches had demonstrated the link between PAAs and the inflammation which were brought by tissue damage.^[18]^ But in this analysis, minimally invasive surgery in studies^[2–4,8–10]^ didn’t reveal a reduced incidence of PAAs, compared with open surgery which paired with more tissue trauma, indicated that traumatic inflammation might only act a relatively smaller part on AAs after esophagectomy.

The impact of AAs after esophagectomy on mortality was in dispute. Study^[3,7,11]^ found no influence on perioperative and 60-day mortality while others^[2,8–10]^ indicated the contrary. With combined evidence in this meta-analysis, we found that AAs after esophagectomy were associated with higher perioperative mortality but no definitive conclusion with 60-day mortality. PAAs might be surrogate for perioperative mortality, since no death was directly attributed to it. Underlying mechanism for the association, by speculating, could be that PAAs resulting in hypoperfusion and more postoperative complications. The meta-analysis showed AAs following esophagectomy were associated with more events in complications like pulmonary pneumonia, anastomotic leakage, and possibly renal failure. Most PAAs occurred within 3 days after esophagectomy. From perspective of the temporal relationship, pulmonary pneumonia preceded with the onset of PAAs while diagnosis of anastomotic leak was made days after the onset of AF. PAAs leading to hypoperfusion may be the explanation for the association to anastomotic leak, and anastomotic leak from anatomical adjacent could in turn stimulate the atria into PAAs. The relation between pulmonary pneumonia and PAAs was well established.^[19,20]^ High events of complications associated with PAAs would inevitably extend the hospital and ICU stay and longer hospital stay could reciprocally increase the incidence of complications.

Treatment strategies varied among studies^[3,4,6,8–11]^ including electrical cardioversion, antiarrhythmic and analgesia medication.^[9]^ The prognosis after antiarrhythmic medication remained in disparity.^[3,6–10]^ Unfortunately, management of PAAs after esophagectomy wasn’t well established. Considering the arrhythmogenic effect, medical intervention should be individualized. Furthermore, towards patients with AAs after esophagectomy, surgeons should be aware of potential morbidity of anastomotic leak and take the initiative to go through a screen test and deliver treatment when the diagnosis has been confirmed.

The major limitation of this meta-analysis is heterogeneity which is generated by the retrospective, observational nature of the studies. Measured by *I*^2^ values, heterogeneity was detected in 60-day mortality, and renal failure, which we take the random effect model into meta-analysis with. Two studies^[5,10]^ with the same corresponding author had overlapping time interval, raising the potential overlapping of patients populations, which was dealt with eliminating the former one.

This meta-analysis has shown that PAAs after esophagectomy is a common complication, which is associated with increased perioperative mortality; longer hospital stay and high prevalence of postoperative complications. Further research should be focusing on prophylaxis and developing standardized treatment.

## Author contributions

**Conceptualization:** Lai-Te Chen.

**Software:** Lai-Te Chen.

**Data curation:** Lai-Te Chen.

**Formal analysis:** Lai-Te Chen.

**Investigation:** Lai-Te Chen.

**Methodology:** Lai-Te Chen.

**Project administration:** Lai-Te Chen.

**Resources:** Lai-Te Chen, Chenyang Jiang.

**Software:** Lai-Te Chen.

**Supervision:** Chenyang Jiang.

**Validation:** Lai-Te Chen.

**Visualization:** Lai-Te Chen.

**Writing – original draft:** Lai-Te Chen.

**Writing – review & editing:** Lai-Te Chen.
